# Dysregulation of the Mammalian Target of Rapamycin and p27^Kip1^ Promotes Intimal Hyperplasia in Diabetes Mellitus

**DOI:** 10.3390/ph6060716

**Published:** 2013-05-27

**Authors:** Thomas Cooper Woods

**Affiliations:** 1 Tulane Heart and Vascular Institute and the Department of Physiology, School of Medicine, Tulane University, 1430 Tulane Avenue, SL-48, New Orleans, LA 70112, USA; E-Mail: twoods3@tulane.edu; Tel.: +1-504-988-2588; Fax: +1-504-988-2675; 2 Department of Pharmacology & Experimental Therapeutics, LSU Health Sciences Center—New Orleans, New Orleans, LA 70112, USA

**Keywords:** intimal hyperplasia, p27^Kip1^, mammalian Target of Rapamycin, diabetes mellitus, vascular smooth muscle cells

## Abstract

The proliferation and migration of vascular smooth muscle cells (VSMCs) in the intima of an artery, known as intimal hyperplasia, is an important component of cardiovascular diseases. This is seen most clearly in the case of in-stent restenosis, where drug eluting stents are used to deliver agents that prevent VSMC proliferation and migration. One class of agents that are highly effective in the prevention of in-stent restenosis is the mammalian Target of Rapamycin (mTOR) inhibitors. Inhibition of mTOR blocks protein synthesis, cell cycle progression, and cell migration. Key to the effects on cell cycle progression and cell migration is the inhibition of mTOR-mediated degradation of p27^Kip1^ protein. p27^Kip1^ is a cyclin dependent kinase inhibitor that is elevated in quiescent VSMCs and inhibits the G_1_ to S phase transition and cell migration. Under normal conditions, vascular injury promotes degradation of p27^Kip1^ protein in an mTOR dependent manner. Recent reports from our lab suggest that in the presence of diabetes mellitus, elevation of extracellular signal response kinase activity may promote decreased p27^Kip1^ mRNA and produce a relative resistance to mTOR inhibition. Here we review these findings and their relevance to designing treatments for cardiovascular disease in the presence of diabetes mellitus.

## 1. Introduction

Cardiovascular diseases initiate with an initial insult to a healthy artery that elicits an inflammatory response [1,2,3,4]. This inflammatory response results in the development of a plaque through the recruitment of inflammatory cells to the site of injury and the proliferation and migration of vascular smooth muscle cells (VSMC) in the intimal layer of the artery wall. Continued development of this plaque leads to a narrowing of the vessel and a reduction of blood flow. Ultimately, the plaque may become unstable and rupture leading to myocardial infarction or stroke. The proliferation and migration of VSMCs, known as intimal hyperplasia, is also a limiting factor in the use of stents for the prevention of restenosis following balloon angioplasty. Inhibitors of the mammalian Target of Rapamycin (mTOR) are highly effective at blocking intimal hyperplasia and have been used in drug-eluting stents. This review will focus on the regulation of intimal hyperplasia and potential changes in the role of the mTOR pathway in intimal hyperplasia in the presence of diabetes.

## 2. Role of the Cyclin-Dependent Kinase Inhibitor, p27^Kip1^, in Cardiovascular Disease

### 2.1. The Vascular Response to Injury

Vascular diseases arise from an initial insult or injury to the vessel [1,2,3,4]. This injury can be mechanical or biological in nature. Mechanical injury includes balloon dilatation and endothelial disruption during percutaneous coronary angioplasty as well as turbulent flow or oscillatory shear stress. The most studied biological example would be the formation of fatty streaks early in atherosclerosis, but biological injury also includes excess free radicals, viral infection, and aspects of diabetes [5,6,7]. Despite differing causes, the result is the same, the loss of the integrity of the endothelial lining of the artery wall and an increase in the expression of adhesion molecules on the endothelial surface that promote the recruitment of leukocytes to the site of injury, initiating an inflammatory process. Leukocytes migrate into the medial layer of the vessel and release cytokines and growth factors that serve to amplify the inflammatory response [8,9]. This leads to an induction of inflammatory gene expression throughout the artery wall, a loss of normal vasofunction, and expression of matrix metalloproteases. The medial vascular smooth muscle cells (VSMCs) respond to these events by migrating to the intima and proliferating. This process, called intimal hyperplasia, results in the formation of a neointima that reduces luminal area.

The vasoactive compounds that promote the vascular response to injury are diverse. E- and P-selectin, vascular cell adhesion molecule-1 (VCAM-1), and intercellular adhesion molecule-1 (ICAM-1) are expressed early at sites of endothelial distress driving the recruitment of leukocytes [10,11,12,13]. Cytokines, such as monocyte chemotactic protein-1 and the interleukin family, and growth factors, such as platelet derived growth factor (PDGF), basic Fibroblast Growth Factor (bFGF), Angiotensin II (Ang II), and vascular endothelial cell growth factor (VEGF), promote the diapedesis of monocytes into the medial layer of the artery as well as the migration of VSMCs to the intimal layer where they proliferate to produce a neointima [4,14,15]. Thus, a diverse set of molecules serve to promote VSMC proliferation and migration through varied pathways, diminishing the effectiveness of targeting a single receptor or ligand. As we discuss below, targeting processes integral to proliferation and migration (e.g., cell cycle progression) has proven effective in preventing intimal hyperplasia.

### 2.2. Intimal Thickening is Blocked by Elevated Levels of the Cyclin Dependent Kinase Inhibitor, p27^Kip1^

As VSMCs exit quiescence, p27^Kip1^ is down-regulated through translocation to the cytoplasm, in part, facilitated by phosphorylation at its serine 10 (S10) residue [16]. In the cytoplasm, it is ubiquitinylated by the E3-ubiquitin ligase complex KPC1/2 and degraded by the proteasome [17,18,19]. Later in the cell cycle, p27^Kip1^ is phosphorylated at the threonine 187 (T187) residue forming a docking site for a second E3-ubiquitin ligase which includes Skp2, again resulting in its ubiquitinylation and degradation by the proteasome [20,21,22]. The decrease in p27^Kip1^ protein levels releases cyclin E/cyclin-dependent kinase 2 (cdk2) complexes to hyperphosphorylate the retinoblastoma protein (pRb) resulting in the transcription of genes required for the G_1_-S transition [23,24].

Beyond cell cycle regulation, p27^Kip1^ has also been shown to regulate migration. Cellular migration requires the formation of a gradient in the activities of the small GTPases Rho and Rac [25]. Elevated Rac activity promotes cellular protrusions at the leading edge of migration, while Rho activation maintains cellular adhesion to achieve traction. In VSMCs, pharmacological inhibition of Rho activation inhibits cellular migration [26]. Elevated levels of cytoplasmic p27^Kip1^ has also been shown to regulate migration directly through its ability to bind and block activation of RhoA [27,28].

Initial studies in cultured VSMCs and in the porcine model of vascular injury suggested a critical role for the down regulation of p27^Kip1^ via activation of mTOR in the progression of intimal hyperplasia [29,30,31,32,33]. Elevated p27^Kip1^ inhibits vascular cell proliferation and migration [27,34]. A follow-up report, using a global p27^Kip1^ knockout mouse, suggested that p27^Kip1^ deficiency did not alter intimal hyperplasia or the effects of mTOR inhibition [35]. However, recent reports demonstrate a central role for the Skp2 mediated degradation of p27^Kip1^ protein in the vascular response to injury. p27^Kip1^ levels were increased and intimal hyperplasia reduced both in Skp2-/- mice following carotid ligation and in balloon injured rat carotids treated with an adenovirus expressing a dominant negative Skp2 [36]. Likewise, deletion of AMPKα2 exacerbates intimal hyperplasia through an increase in Skp2 and a decrease in p27^Kip1^ [37]. In addition to p27^Kip1^, inhibition of mTOR leads to an increase in another target of Skp2, the cyclin dependent kinase inhibitor, p21^Cip^, in VSMCs [38]. Increased p21^Cip^ in VSMCs is associated with reduced VSMC proliferation and inhibition of intimal hyperplasia, suggesting a potential mechanism for rapamycin’s effectiveness in the p27Kip1 null mouse [39,40]. With respect to atherosclerosis, several reports have found that loss of p27^Kip1^ serves to accelerate atherosclerosis [41,42] or that increased p27^Kip1^ retards plaque formation [43]. These reports highlight the critical role of p27^Kip1^ in intimal hyperplasia and atherosclerosis.

## 3. Clinical Use of mTOR Inhibitors in the Treatment of Cardiovascular Disease

The introduction of drug eluting stents that deliver the mTOR inhibitor, rapamycin, greatly reduced the intimal hyperplasia component of in stent restenosis demonstrating a critical role for this pathway in the vascular response to injury [44]. mTOR is a phosphatidylinositol-related kinase that regulates cell growth and proliferation in response to mitogens and nutrients through regulation of translation, transcription, and cell cycle progression. mTOR forms two functionally distinct complexes. The mTOR complex 1 (mTORC1) consists of mTOR, the rapamycin sensitive adapter protein of mTOR (Raptor), and mLST8 (also known as GβL). This complex regulates ribosomal biogenesis and protein synthesis through activation of p70^S6kinase^ to initiate ribosomal S6 kinase and inhibition of the 4E-binding protein-1’s (4E-BP-1) ability to restrict mRNA translation [45,46]. Additionally, inhibition of p70^S6kinase^ by rapamycin promotes expression of smooth muscle contractile proteins, suggesting inhibition of the mTOR/p70^S6kinase^ pathway also promotes VSMC differentiation [38]. The mTOR complex 2 (mTORC2) is defined by the presence of the rapamycin insensitive companion of mTOR (rictor), the SAPK-interacting protein (SIN1), and mLST8. mTORC2 is linked to actin cytoskeleton regulation and phosphorylation of the protein kinase Akt (also known as Protein Kinase B) [47,48,49,50,51]. Rapamycin and its analogs bind to FK506 binding protein-12 (FKBP12) and inhibit mTORC1. Though the rapamycin-FKBP12 complex cannot bind mTORC2, prolonged exposure to rapamycin blocks formation of nascent mTORC2 resulting in inhibition of its downstream effects [48]. Treatment with rapamycin also leads to an increase in the protein levels of the cyclin dependent kinase inhibitor, p27^Kip1^, and VSMCs lacking p27^Kip1^ exhibit a relative resistance to rapamycin [31].

Restenosis is the primary limitation to the use of percutaneous transluminal coronary angioplasty (PTCA) and stent implantation in the treatment of coronary artery disease. Despite major advancements, including the use of anti-platelet and anti-thrombotic therapies, in-stent restenosis rates range from 15–20% in treating ideal lesions to 30–60% in treating the more complex lesions [44]. In diabetic patients the restenosis rate is 38–55% after stent implantation [52,53,54,55]. In-stent restenosis results largely from intimal hyperplasia and the use of drug eluting stents (DES) to deliver agents that inhibit VSMC proliferation and migration has proven quite effective in reducing restenosis rates [56]. However, while drug eluting stents are more effective than bare metal stents in preventing in stent restenosis in the diabetic population [57], there is a loss of efficacy in this high risk population [58,59].

## 4. Changes in the Molecular Mechanisms Regulating Cell Proliferation and Migration under Diabetic Conditions

Initial studies into the interplay of diabetes and cardiovascular disease have focused on the role of hyperglycemia [60,61,62], inflammatory mediators [60,63], and reactive oxygen species [64,65,66] on the vasculature. Diabetes is a complex disease and the increase in inflammation and oxidative stress under diabetic conditions clearly promotes increased CVD in the diabetic population [67,68]. However, cellular and molecular changes in the response of VSMCs to injury have not been fully addressed. Recent large clinical studies have demonstrated that intensive control of blood glucose does not by itself reduce CVD events in type 2 diabetics [69,70,71,72,73]. This finding may depend on the strategy used to achieve the glucose control or it may suggest that targeting changes in the vasculature in response to diabetes are necessary to reduce CVD events in this population. We and others have found an increase in the activity of the extracellular signal response kinase 1/2 (ERK) pathway in the vascular tissue of diabetic animal models compared to non-diabetic controls [34,74,75]. Furthermore, our data suggest that the increase in ERK activity leads to a relative resistance to mTOR inhibition [34,76]. Below, we discuss three examples of aspects of type 2 diabetes that may promote increased ERK activity in the presence of diabetes mellitus.

### 4.1. Role of Ang II in VSMC Insulin Resistance

Clinically the use of angiotensin converting enzyme inhibitors and angiotensin receptor blockers has been shown to reduce cardiovascular disease and increase insulin sensitivity [77,78], suggesting a role for Ang II in the insulin resistance of the vasculature. Acute administration of Ang II inhibits the insulin stimulated association of IRS-1 with p85, which subsequently activates Akt mediating the metabolic effects of insulin [63]. Chronic infusion of Ang II has been shown to promote IRS-1 phosphorylation and proteasome dependent degradation in a reactive oxygen species (ROS)-dependent manner [65]. As NADPH oxidase activity and ROS production are increased in the vasculature of type 2 diabetic animals, this may represent a general mechanism that induces insulin resistance in the vasculature. A key element of this effect is that while IRS-1/PI3K/Akt signaling is lost, activation of the ERK signal transduction pathway is maintained. Thus, this mechanism of producing insulin resistance includes a loss of the metabolic effects of insulin while maintaining the pro-atherogenic effects, specifically the IR/Ras/Mek/ERK pathway.

### 4.2. Hyperglycemia and IGF-1 Activation of ERK

As stated above, the mechanisms underlying the increased vascular disease accompanying diabetes is likely multi-factoral. In addition to the effects of elevated ROS production, hyperglycemia has been shown to exhibit multiple inflammatory and pro-atherogenic effects. Hyperglycemia promotes the formation of advanced glycation end products (AGEs) which promote VSMC migration and proliferation through binding to the receptor for AGEs. Elevated glucose levels have also been shown to enhance the proliferation and migration of VSMCs in response to IGF-1 [61,62]. This enhancement occurs through a hyperglycemia induced increase in extracellular matrix production that increases the levels of ligands for integrin αVβ3 and permits phosphorylation of the adaptor protein, Shc, in response to IGF-1 stimulation [61,62]. The addition of Shc phosphorylation augments the activation of the ERK pathway in response to IGF-1 stimulation.

### 4.3. Changes in Insulin Signaling in Response to Changes in IGFR Expression

VSMCs express both the IR and IGFR, with the latter being expressed at approximately eight fold higher levels [79,80,81]. As hybrid receptors are formed randomly according to the ratio of proreceptors [82,83,84], many of the IR heterodimers may function as half of a hybrid receptor. The hybrid receptors exhibit similar affinity for insulin as the IGFR [85,86]. IGFR levels are dynamically regulated and responsive to physiological stimuli with decreased expression occurring in response to IGF-1 [87]. Overexpression of IGFR in VSMCs results in a loss of Akt phosphorylation in response to 7 nM insulin (a concentration which did not activate the IGFR) [87]. In contrast, down-regulation of the IGFR increased phosphorylation of Akt and ERK in response to insulin [87]. This suggests that changes in the expression of the IGFR and subsequent changes in the number of IR holoreceptors may increase ERK activation.

Overall, a common factor among all of these aspects of type 2 diabetes is either a direct or indirect promotion of ERK activation. These examples represent a larger list of changes in the regulation of ERK and other pathways that may alter the regulation of proliferation and migration in the presence of diabetes. While activation of ERK is known to promote VSMC proliferation and migration directly, the impact of increased activation of ERK on other pathways that normally dominantly regulate intimal hyperplasia is not known.

**Figure 1:**
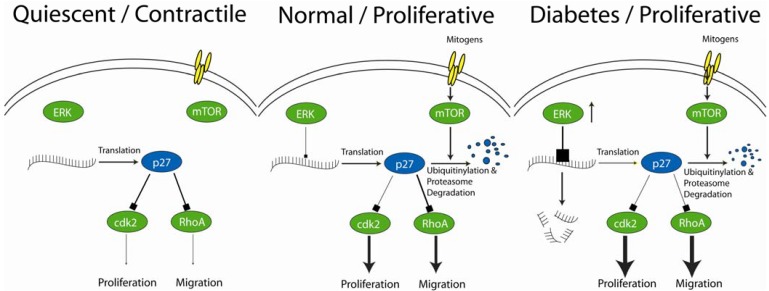
Under quiescent conditions, p27^Kip1^ levels are high inhibiting cdk2 and RhoA activity thereby blocking VSMC proliferation and migration (Left Panel). In normal VSMCs, mitogenic stimulation promotes the proteasome dependent degradation of p27^Kip1^ protein through, at least in part, activation of the mTOR pathway (Center Panel). Loss of p27^Kip1^ permits increased activation of cdk2 and RhoA leading to VSMC proliferation and migration. In the presence of diabetes, ERK activity is increased, which in turn destabilizes p27^Kip1^ mRNA (Right Panel). This lowers p27^Kip1^ protein levels before mitogenic stimulation occurs, allowing for greater proliferation and migration. Activation of the mTOR pathway functions as it did under normal conditions and will promote down regulation of p27^Kip1^ protein as before.

## 5. Effects of Diabetes Mellitus on Efficacy of mTOR Inhibitors in Preventing In-Stent Restenosis

In two separate VSMC models of diabetes, our lab has found a relative resistance to rapamycin’s effects on proliferation and migration. First, VSMCs isolated from a mouse expressing the IR in only the brain and liver exhibit a diabetic phenotype that includes increased VSMC proliferation and migration [34]. These cells also exhibit a relative resistance to the antiproliferative effects of mTOR inhibition that is abolished with ERK pathway inhibition. Rapamycin treatment of these cells leads to inhibition of p70^S6kinase^ phosphorylation, but does not lead to an increase in p27^Kip1^ protein levels. This loss of p27^Kip1^ is derived from a ERK-dependent decrease in the half-life of p27^Kip1^ mRNA [34]. Our work in endothelial cells suggests that rapamycin blocks p27^Kip1^ degradation through inhibition of phosphorylation of p27^Kip1^ at the Skp2 recognition site (threonine 187) [27]. This suggests that the relative resistance to mTOR inhibition by rapamycin is derived from repression of p27Kip1 at the mRNA level, thereby preceding the effects of mTOR on p27Kip1 protein. In a similar manner, human coronary artery smooth muscles cells obtained from diabetic donors exhibit increased proliferation and migration rates that were resistant to inhibition by rapamycin [76]. These VSMCs also exhibited a similar dysregulation of mTOR and p27Kip1 coupled with an increase in the activity of the ERK pathway.

Together these data suggests a working model for the acceleration of intimal hyperplasia and development of a relative resistance to mTOR inhibition through increased activation of the ERK pathway under diabetic conditions (Figure 1). Under normal conditions, mitogenic stimulation of VSMCs results in cell cycle progression, proliferation, and migration through down regulation of p27^Kip1^. In the presence of diabetes mellitus, ERK activity is increased, destabilizing p27^Kip1^ mRNA and abolishes the check on VSMC proliferation and migration under basal conditions. The end result is an increase in intimal hyperplasia and a diminishing of the role of mTOR in VSMC proliferation and migration.

## 6. Conclusions

Clinically, rapamycin and its analogs are highly effective in preventing in-stent restenosis and remain the best option for the prevention of in stent restenosis. However, there is growing evidence that type 2 diabetes is accompanied by activation of ERK and other pathways. This increase in ERK activity may, in addition to its normal pro-atherosclerotic functions, diminish the role of other pathways that are normally dominant in proliferation and migration. Inhibition of these additional pathways in high risk populations may be necessary to restore the effectiveness of broad based therapeutics.
